# Multisensory inclusive design with sensory substitution

**DOI:** 10.1186/s41235-020-00240-7

**Published:** 2020-08-08

**Authors:** Tayfun Lloyd-Esenkaya, Vanessa Lloyd-Esenkaya, Eamonn O’Neill, Michael J. Proulx

**Affiliations:** 1 Crossmodal Cognition Lab, University of Bath, Bath, BA2 7AY UK; 2 Department of Computer Science, University of Bath, Bath, UK; 3 Department of Psychology, University of Bath, Bath, UK

**Keywords:** Inclusive design, Inclusion, Design for all, Universal design, Sensory substitution, Cross-modal cognition, Multisensory perception, Cross-modal displays, Human-computer interactions

## Abstract

Sensory substitution techniques are perceptual and cognitive phenomena used to represent one sensory form with an alternative. Current applications of sensory substitution techniques are typically focused on the development of assistive technologies whereby visually impaired users can acquire visual information via auditory and tactile cross-modal feedback. But despite their evident success in scientific research and furthering theory development in cognition, sensory substitution techniques have not yet gained widespread adoption within sensory-impaired populations. Here we argue that shifting the focus from assistive to mainstream applications may resolve some of the current issues regarding the use of sensory substitution devices to improve outcomes for those with disabilities. This article provides a tutorial guide on how to use research into multisensory processing and sensory substitution techniques from the cognitive sciences to design new inclusive cross-modal displays. A greater focus on developing inclusive mainstream applications could lead to innovative technologies that could be enjoyed by every person.

## Significance

Sensory substitution devices transform the representation of one sensory input into a new representation with a different sensory form. For example, a visual feed from a camera can be turned into sound to be heard or into tactile stimuli that can be felt. The most common applications of this to date are in developing assistive technologies which aid vestibular problems, visual impairments and hearing impairments. State-of-the-art sensory substitution techniques can contribute significantly to our understanding of how the brain processes and represents sensory information. This progressively advances cognitive theories with respect to multisensory perception and cognition, attention, mental imagery and brain plasticity. Sensory substitution techniques provide a novel opportunity to dissociate the stimulus, the task and the sensory modality, and thus offer a unique way to explore the level of representation that is most crucial for cognition. Due to their versatility, sensory substitution phenomena have the potential to help translate the principles underpinning cognitive theories of multisensory perception into other interdisciplinary research areas, such as human-computer interaction and artificial intelligence. In this review, we provide a novel framework which has two main aims: (i) to explain how applying sensory substitution techniques in a multisensory context for inclusive design can have a wide benefit to society beyond individuals with disabilities and (ii) to explain how inclusive cross-modal displays which utilise sensory substitution techniques will contribute to future cognitive theories of sensory processing.

## Introduction

Disability has previously been regarded as something which professionals should seek to cure and attempt to provide rehabilitation for, enabling individuals to make strides towards a more ‘normal’ existence (Dewsbury, Clarke, Randall, Rouncefield, & Sommerville, 2004). This view of disability has been rejected by disability rights activists, and today, barriers created by society are more commonly seen as being disabling for those with ‘impairments’ (Oliver, 2013). In this review, we argue that new cross-modal displays should be developed which target a mainstream audience and have an inclusive design. This approach to design is about creating products or services which address the needs of the widest possible audience and can therefore be used by anyone, regardless of their age or abilities (Design Council, 2019). We focus specifically on sensory substitution techniques, which build on cognitive mechanisms to represent one sensory form with an alternative and can be utilised to develop inclusive cross-modal displays.

### What does it mean for design to be inclusive?

The social model of disability arose following the Civil Rights Movement during the 1950s and 1960s (Gallagher, Connor, & Ferri, 2014). This model rejects the idea that disability should be viewed as a personal tragedy, a so-called medical model of disability and, instead, emboldens those who have impairments to demand that disabling barriers in society are dismantled (Burchardt, 2004). Coinciding with this change in perspective of disability rights has been a shift within design and engineering to inventing products and services which fulfil the needs of all users, regardless of any impairments. This is known as inclusive design, which is also termed Design for All (within Europe) and Universal Design (in Japan and America). Inclusive design seeks to remove barriers that people with different levels of capabilities may encounter by keeping potential barriers in mind at every stage of the design process (Newell, 2003).

For mainstream products to have an inclusive design, they should either have the ability to cater to all users without the need for any modification or adaptation or they should have the capacity for specialised access equipment to be attached to the product, giving the original product greater functionality (Newell, 2003). Currently, few design courses teach a social model of disability, and perhaps as a result of this, many mainstream products continue to overlook the needs of individuals with impairments (Gieben-Gamal & Matos, 2017). People who have impairments are continually viewed as somewhat of a niche population, and separate products, often called assistive technologies, are used to support them (Gieben-Gamal & Matos, 2017). However, importantly, good inclusive design which improves access to those with impairments can bring benefits to everyone. When products are made which overcome barriers faced by a subset of users, their effectiveness for all users is often improved (Persson, Åhman, Yngling, & Gulliksen, 2015).

That no design can realistically meet the needs and desires of every individual in the population is widely accepted (Bichard, Coleman, & Langdon, 2007). However, when inclusive designs consider the users to be consumers or customers, a competition between different designs to become the most desirable product will ultimately grow, and this will result in a variety of products to suit different preferences (Newell, 2003). The creation of a diversity of inclusive products provides users who have impairments with the freedom to make choices about the ways in which they would like to engage with the environment.

Future technologies could benefit from applying research from the cognitive sciences to new devices which can be enjoyed by everyone. Embedding cognitive theories into design principles can improve how we build inclusive technologies and interact with them (Obrist, Gatti, Maggioni, Vi, & Velasco, 2017; Oviatt, 1999). This paper presents the argument that the development of new cross-modal displays with multisensory modes for the mainstream will serve to benefit all users, regardless of any impairments they might have.

### Crossing the boundaries from cognitive science to human computer interaction research

The current paper will demonstrate how cognitive theories can contribute to shaping future technologies. We provide a tutorial guide on how to use research into multisensory processing to design new inclusive cross-modal displays. We first provide an overview of the concepts surrounding multisensory processing and outline the three guiding principles which are necessary for multisensory processing to occur. Next, we give an overview of the possible outcomes which can be achieved by a cross-modal display and explain how these differ from one another. We provide an overview of sensory substitution techniques including how they work and how they have been implemented. We then explain how sensory substitution techniques can be utilised for the purposes of creating inclusive technologies. Finally, we suggest a number of future applications of inclusive cross-modal displays, which everyone, regardless of ability, could enjoy.

## Understand the literature on multisensory processing

Gaining an overview of the key terms used in the literature and the key principles underlying multisensory perception are important when building an inclusive cross-modal display. In this section, we provide a framework for multisensory processing by defining the key terms used by researchers in this area. We then describe the guiding principles underlying multisensory processing.

### Framework for multisensory processing

Multisensory perception is where information from more than one sense is processed simultaneously. The terminology associated with multisensory perception can be inconsistent and confusing, and the same terms are sometimes used by cognitive scientists, computer scientists, applied researchers and others to describe different concepts. Stein et al. (2010) provide a useful guideline for a common nomenclature for multisensory phenomena. To deal with the semantic inconsistencies, they suggest using the generic term ‘multisensory processing’ to describe any multisensory phenomena such as multisensory integration or multisensory combination. Table 1 further defines some of the key terms used to describe concepts associated with multisensory processing. Recognising that these concepts vary in the properties to which they have been attributed is important. Some of the concepts refer to a neural or behavioural response, some a display type and some a sensory source (Table 1). For example, the term ‘multisensory’ refers to the internal neural and behavioural response when multiple senses are stimulated. This is fundamentally distinct from the term ‘cross-modal’, which refers to the external sensory source in the environment which emits information that can be processed by more than one sense.

**Table 1:** Definitions of key terms from cognitive neuroscience studies to describe concepts relating to multisensory processing, as outlined by Stein et al., 2010

Term	Definition	Property the concept is attributed to
Unisensory	Any neural or behavioural process associated with a single sense	Neural or behavioural responses
Multisensory	Any neural or behavioural process associated with multiple senses	Neural or behavioural responses
Cross-modal display mode	A display with multiple display modes to channel sensory information of different origins	Display type
Multisensory integration	A specific multisensory processing where redundant sensory information is optimally integrated to result in a multisensory response significantly different than their unisensory correspondence	Neural or behavioural response
Multisensory combination	A specific multisensory process where complementary sensory information is combined to result in a more accurate estimate of the sensory source	Neural or behavioural response
Modality-specific	The sensory information from a source that results in a unisensory response	The sensory source
Cross-modal	The sensory information from a source that results in a multisensory response	The sensory source
Spatial coincidence	The spatial overlap between two or more cross-modal stimuli of the same sensory source	The sensory source
Temporal coincidence	The temporal overlap between two or more cross-modal stimuli of the same sensory source	The sensory source
Redundancy	The reliability of spatially and temporally overlapping cross-modal stimuli of the same sensory source	The sensory source, and the neural and behavioural response
Complementary	Sensory information from the same sensory source that is not spatially or temporally overlapping	The sensory source, and the neural and behavioural response
Inverse effectiveness	The influence of reliability on cross-modal cues in multisensory processing	Neural or behavioural response
Cross-modal correspondence	Associations between different sensory forms	Neural or behavioural response

There are three guiding principles which are necessary for multisensory processing to occur: spatial coincidence, temporal coincidence and inverse effectiveness (Kayser & Logothetis, 2007). We next provide an overview of each of these principles.

### Principles of spatial and temporal coincidence

In order for multisensory processing to happen, cross-modal information needs to come from spatially aligned sensory sources (Stein, 1998). This is known as the spatial coincidence principle. Cross-modal information also needs to come from sources which are in close temporal proximity to one another (Stein & Wallace, 1996). This is known as the temporal coincidence principle. For example, we can see spatial and temporal coincidence failing when technical glitches cause actors’ speech to become asynchronous to their lip movements. In this case, the auditory information is not aligned with the visual information. Cross-modal information that is not spatially or temporally aligned may be perceived as if it comes from separate sensory sources, causing a depression in the multisensory response and instead leading to separate unisensory responses (Stein, 1998; Stein & Wallace, 1996).

Principles of spatial and temporal coincidence are manipulated in a number of human-computer interaction (HCI) studies to investigate the margins between congruent and incongruent cross-modal cues for improving user experience and performance. For example, congruent visual and audio/tactile stimuli can be used to increase the perceived quality of buttons on touch-screen devices (Hoggan, Kaaresoja, Laitinen, & Brewster, 2008). HCI researchers also make use of the principle of spatial and temporal coincidence by purposefully utilising incongruent multisensory stimuli in some technologies. For example, one limiting factor of current virtual reality technologies is the way in which users underestimate the distance between themselves and the target object, known as distance compression. To manage distance compression, incongruent audio-visual stimuli can be designed to artificially align the two senses (Finnegan, O’Neill, & Proulx, 2016).

### Principle of inverse effectiveness

For multisensory processing to happen, the cross-modal stimuli which are compared with one another need to be approximately equally reliable. If a cue from one modality elicits a stronger behavioural response than the cue from another modality when presented together from the same sensory source, the multisensory processing of the sensory source will be weakened (Perrault, Vaughan, Stein, & Wallace, 2003; Stanford, 2005; Stanford & Stein, 2007; Stein & Wallace, 1996). This is known as the inverse effectiveness principle. The multisensory calibration, and thus the reliability, of our senses usually emerges at different critical periods during development (Bremner, 2017). For example, children are thought not to become optimally proficient in integrating visual and tactile information until at least the age of eight (Gori, Del Viva, Sandini, & Burr, 2008; Nardini, Jones, Bedford, & Braddick, 2008; Scheller, Proulx, de Haan, Dahlmann-Noor, & Petrini, 2020). Similarly, acquired senses (e.g., via sensory augmentation devices) can be calibrated with intact senses and adjusted for multisensory processing through learning and experience, thereby increasing their reliability (Proulx, Brown, Pasqualotto, & Meijer, 2014). The principle of inverse effectiveness also gives rise to the phenomenon that users of augmentation displays must rely heavily on their intact senses until the newly acquired sense becomes equally reliable. Furthermore, if more than one acquired sense is utilised via sensory augmentation devices, their reliability is expected to be equal to each other because they are both unfamiliar to the user (Proulx et al., 2014).

## Determine the possible outcomes for cross-modal displays

When building an inclusive cross-modal display, gaining a comprehensive understanding of the possible outcomes from the device is important. Two possible outcomes exist from cross-modal displays: multisensory integration or multisensory combination (Table 1). Multisensory integration and multisensory combination are two of the terms which are used inconsistently in the literature (Stein et al., 2010). Applied researchers tend to use the term multisensory integration, but the use of this term is sometimes misleading. Fundamental differences exist between these outcomes, which require some explanation to prevent misunderstandings occurring later in the design process. Therefore, we next provide an explanation of these two separate outcomes.

### Multisensory integration

Multisensory integration is where cross-modal cues are integrated to give a perception which is significantly different from the perception experienced when only one cue is processed (Stein et al., 2010). We can conceptualise this by imagining how we might perceive a piece of fruit, such as a pear. If we only see the pear, we might say its size is approximately 6 units. However, we might perceive the pear to be slightly bigger, perhaps 8 units, if we were given the pear to hold with our eyes closed. If we hold the pear while looking at it, assuming both senses are equally reliable, the size of the pear would be perceived to be approximately 8 units. Multisensory integration happens when both senses are used to inspect the pear, reducing uncertainty regarding its size and giving an estimate that is somewhere between each individual unisensory estimate (Rock & Victor, 1964).

Multisensory integration is viewed as the neural process of integrating redundant sensory cues in an optimal fashion (Rohde, van Dam, & Ernst, 2016). Here, the level of redundancy is a function of the principles of spatial and temporal coincidence and of inverse effectiveness. By this definition, a high level of redundancy occurs when the reliability of multiple senses is approximately equal and when the sensory sources are spatially and temporally aligned. Thus, in our pear example, a high level of redundancy is present. To perceive the pear accurately as 7 units, we rely equally on our vision and our touch. The level of redundancy can change when environmental circumstances temporarily reduce the reliability of one or more of our senses. For example, when the pear is inspected under a magnifying glass, the reliability of our vision decreases to estimate its real size. When the discrepancy between the reliability of different senses becomes higher, the level of redundancy decreases. When redundancy is too low, no multisensory integration occurs. For example, if we were to hold the pear while looking at it under a magnifying glass, our tactile perception would still suggest it has a size of 8 units, yet our visual perception might suggest it has a size of 20 units. An automatic process is thought to happen during multisensory integration, whereby greater statistical weight is assigned to the more reliable sensory source (Talsma, Senkowski, Soto-Faraco, & Woldorff, 2010). Thus, when magnification is applied, more weight is assigned to our tactile sense, and the high discrepancy in reliability means redundancy becomes too low for multisensory integration to occur. Rather than a multisensory percept, we perceive the visual and auditory information as unisensory. Since more weight is assigned to our tactile sense, we perceive the pear to have a size of 8 units.

### Multisensory combination

Multisensory combination is another possible outcome of multisensory processing. This again provides a more accurate estimation of something in space, such as an object, compared to unisensory perception, but the process to arrive at this estimation is fundamentally different from the process of multisensory integration. In this case, the perception we experience from one sense provides complementary information to the perception derived from another sense. The two experiences are combined to give a more robust estimation of the sensory source (Bülthoff & Mallot, 1988). Using the pear example, when we see the pear from the front we might perceive the pear to be approximately 6 units. Next, we pick the pear up and we can feel a bulge on the back of the pear. We could not tell from looking at the pear that its shape is asymmetrical and its back is much more convex than its front; this information is not redundant. This new information causes us to change our perception of the size of the pear (Newell, Ernst, Tjan, & Bülthoff, 2001). We now know that the pear must be bigger than 6 units, therefore our multisensory perception has provided a more accurate estimate than our visual perception alone could provide.

**Fig. 1:**
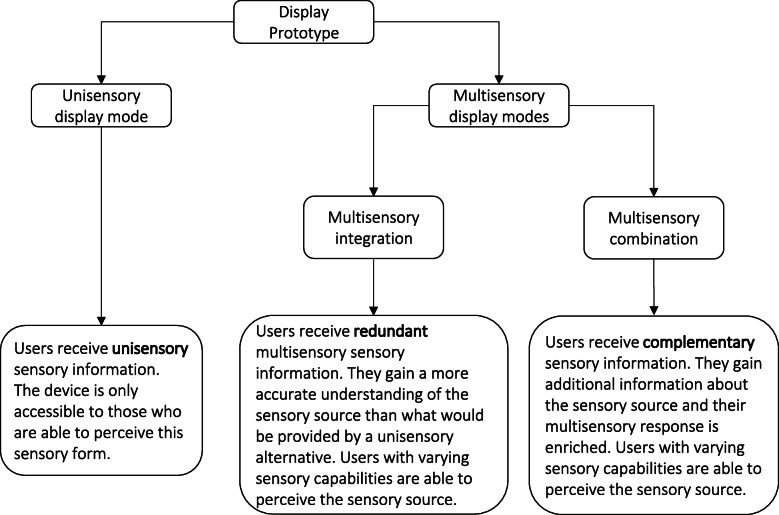
Illustration of how unisensory and cross-modal display modes can utilise various sensory processing

A summary to show how the outcomes of multisensory processing can be applied when prototyping cross-modal display modes is provided in Fig. 1. Cross-modal displays can either achieve multisensory integration, which provides the user with redundant sensory information. This enables the user to use multiple senses to acquire a more accurate estimate of the sensory source than would be achieved by a unisensory alternative. In achieving multisensory combination, on the other hand, cross-modal displays are not limited by providing redundant information. Instead, they enable the user to use multiple senses to gain additional information which has the effect of maximising what they can perceive from the environment.

Most studies within HCI research aim to achieve multisensory integration (Stein et al., 2010). While multisensory integration is a common natural phenomenon, such integration is very difficult to achieve using artificial devices such as sensory augmentation devices. This is due in part to the length of time that is necessary for adaptation. Spatially and temporally aligning sensory information to resemble a natural sensory source is also challenging in digital environments. Few HCI researchers refer to multisensory combination. However, some have used complementary cross-modal cues to decrease user’s cognitive loads in mobile devices (Hoggan & Brewster, 2007). While not mentioned explicitly, this is an example of multisensory combination because the perceived experience of touchscreen buttons were enhanced with the combination of visual and complementary audio/tactile feedback (Hoggan et al., 2008). A gap currently exists between cognitive theory and applied research because HCI researchers find it difficult to demonstrate that devices are effective in achieving multisensory integration. However, a greater focus on multisensory combination could enable applied researchers to evidence the effectiveness of their devices more easily. We therefore recommend that applied researchers should aim for multisensory combination when building new inclusive multisensory devices.

## Utilising sensory substitution techniques to design inclusive cross-modal displays

The cross-modal displays that we are particularly interested in, when thinking about how to create inclusive technology, utilise sensory substitution techniques. These devices have the potential to be used by the mainstream, to enhance any users’ capabilities to interact with their environment. Our next section explains how sensory substitution techniques work, who they are currently used by, and the potential they have for expansion into a broader market.

### The mechanisms underpinning sensory substitution techniques

A single physical feature in the environment clearly can be processed by multiple senses. For example, the edge of a cup can be seen and also touched. Traditionally, it was thought that the brain consisted of independent unisensory modules which processed information before multisensory percepts occurred through bottom-up facilitation only (Choi, Lee, & Lee, 2018). However, this view was later challenged by evidence showing our brains execute metamodal computations and tasks through utilising an integrated network, known as the metamodal organisation of the brain (Pascual-Leone & Hamilton, 2001). This hypothesis takes the view that brain organisation is not necessarily organised by sensory modality but rather by the computational or functional task being carried out (Proulx et al., 2014). For example, seeing and touching the edge of a cup will lead to the activation of multiple senses. Due to the metamodal organisation of the brain, multiple senses will evoke shared cognitive forms to perceive the edge of the cup. The metamodal hypothesis has been repeatedly supported by a growing body of empirical evidence (Brefczynski-Lewis & Lewis, 2017; Ortiz-Terán et al., 2016; Ricciardi, Bonino, Pellegrini, & Pietrini, 2014; Ricciardi & Pietrini, 2011). Indeed, research demonstrates our brains have evolved the ability to use incoming information from multiple senses to create a coherent perception of the environment (Ghazanfar & Schroeder, 2006; Spence, 2011). Furthermore, the bottom-up sensory responses are shown to be modulated by top-down facilitations (e.g., memory and attention) such that previously acquired associations can enhance task-relevant multisensory responses. For a detailed review, see (Choi et al., 2018).

As a result of the metamodal organisation of the brain, sensory information and cognitive forms are learnt and hence gradually associated with one another in relation to bottom-up and top-down facilitations. Since two entirely separate concepts can have shared cognitive forms it is possible for seemingly random concepts to become associated with one another. For example, while an inedible object would not appear to have a taste in one’s mind, research finds that individuals conceive boulders to be sour (Woods, Spence, Butcher, & Deroy, 2013). According to the metamodal theory, this is because boulders and a sour taste are represented by a shared cognitive form in the brain; therefore, the two concepts have become associated. Other unusual findings include lemons being conceived to be fast and prunes to be slow (Woods et al., 2013). Strangely, research has even found both sighted individuals and the early blind, who never experienced colour perception, perceive the colour red to be heavy (Barilari, de Heering, Crollen, Collignon, & Bottini, 2018; Woods et al., 2013). These abstract associations are argued to be the result of shared conceptual dimensions between cognitive forms which are common across cultures and languages (Barilari et al., 2018; Spence, 2011; Spence & Parise, 2012b).

**Fig. 2:**
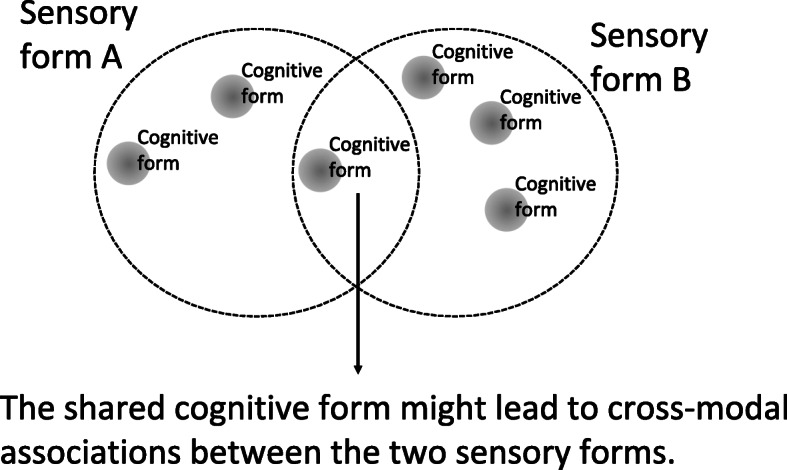
Diagram to show how cross-modal associations can arise when two sensory forms overlap, in line with the metamodal hypothesis. For example, Sensory form A could be pitch (the perception of auditory frequency) and Sensory form B could be visuospatial elevation. The overlapping space would include forms that are “high” such as a high-pitched sound and an object high in elevation (cf. Melara & O’Brien, 1987).

Cognitive scientists have investigated these associations across cultures and languages by looking at the mappings between different sensory forms, which are termed cross-modal correspondences (Spence & Parise, 2012b). An example of cross-modal correspondences is where a higher-pitched signal of an auditory form is associated with a higher vertical elevation of a visual form (Melara & O’Brien, 1987), and a louder sound, with a brighter visual form (Marks, 1974). This means that, due to the metamodal organisation of the brain, stimulating one sense can result in the activation of the same cognitive form that would have been activated when a different sense was stimulated (Fig. 2). The representation of features of a sensory experience, such as seeing, using a different sensory form, such as hearing, is called sensory substitution (Esenkaya & Proulx, 2016). Accordingly, sensory substitution techniques take advantage of cross-modal correspondences by evoking in the brain a cognitive form by stimulating a different sense from the one usually stimulated. Sensory substitution devices (SSDs) are essentially cross-modal displays (Kaczmarek, Webster, Bach-y-Rita, & Tompkins, 1991) which take advantage of the way in which complementary cross-modal cues are associated with one another (Parise & Spence, 2012; Spence, 2011; Spence & Parise, 2012b).

### Sensory substitution devices for assisting those with disabilities

Currently, SSDs are mainly regarded as technology which can assist individuals who have disabilities. Research into SSDs for assisting those who are blind or partially sighted dominates the field, over any other disability. This section provides an overview of the ways in which SSDs work in the context of individuals who are blind and describes current uses of SSDs.

Applying sensory substitution techniques to the visually impaired enables access to visual information via non-visual cross-modal cues (Chebat et al., 2018; Maidenbaum, Levy-Tzedek, Chebat, & Amedi, 2013; Proulx & Harder, 2008). The mappings between visual and auditory forms, in terms of elevation and pitch, and brightness and loudness, can be utilised via SSDs to represent some features of a visual form with an auditory form (Meijer, 1992). In the long term, these pairings may be strongly associated such that late blind people can have visual imagery similar to that of the perception of sight (Esenkaya & Proulx, 2016; Ortiz et al., 2011; Ward & Meijer, 2010). With SSDs, it is possible to acquire visual information by means of sonifications (Meijer, 1992) or two-dimensional tactile cues (Bach-y-Rita & W. Kercel, 2003). It is also possible to acquire auditory information by means of vibrotactile cues (Butts, 2015; Eagleman, Novich, Goodman, Sahoo, & Perotta, 2017).

In developing assistive devices for individuals with visual impairment, research has predominantly investigated visual-to-auditory and visual-to-tactile sensory substitution techniques. For examples of devices, see EyeMusic (Abboud, Hanassy, Levy-Tzedek, Maidenbaum, & Amedi, 2014), Vibe (Durette, Louveton, Alleysson, & Hérault, 2008), See ColOr (Bologna, Deville, & Pun, 2009), The PSVA (Capelle, Trullemans, Arno, & Veraart, 1998), Elektroftalm (Starkiewicz & Kuliszewski, 1963) and the Optophone (d’Albe, 1914). These techniques have been shown to be successful in emotion conveyance, object recognition, localisation, avoidance and navigation tasks (see Table 2 for references). The vast majority of research on visual-to-auditory devices has focused on the associations between the direction of pitch and movement. For some devices, e.g., The vOICe, higher pitched sonification signals are paired with higher elevations of tactile signals. Another device, Synaestheatre, incorporated multiple auditory components and associated these signals with movement. A 3D sensor was used to record depth information using spatialised sounds, enabling azimuth (the horizontal angle) and elevation to be conveyed (Hamilton-Fletcher, Obrist, Watten, Mengucci, & Ward, 2016).

**Table 2:** Table with key references for a variety of cognitive tasks successfully completed by visual-to-tactile or visual-to-auditory sensory substitution techniques

Tasks	Sensory domains	References
Object recognition	Visual-to-auditory	(Auvray, Hanneton, & O’Regan, 2007; Bermejo, Di Paolo, Hüg, & Arias, 2015; Brown, Macpherson, & Ward, 2011; Haigh et al., 2013; Pasqualotto & Esenkaya, 2016; Renier et al., 2005; Stiles & Shimojo, 2015; Stiles, Zheng, & Shimojo, 2015; Striem-Amit, Cohen, Dehaene, & Amedi, 2012)
	Visual-to-tactile	(Akita, Komatsu, Ito, Ono, & Okamoto, 2009; Chebat et al., 2018; Grant et al., 2016; Nau, Bach, & Fisher, 2013; Richardson et al., 2020; Stronks, Mitchell, Nau, & Barnes, 2016)
Localisation	Visual-to-auditory	(Auvray et al., 2007; Borenstein, Ulrich, & Shoval, 2000; Brown et al., 2011; Levy-Tzedek et al., 2012; Pasqualotto & Esenkaya, 2016; Proulx et al, 2010; Stiles et al., 2015)
	Visual-to-tactile	(Akita et al., 2009; Cancar, Díaz, Barrientos, Travieso, & Jacobs, 2013; Chebat et al., 2018; Dublon & Paradiso, 2012; Froese, McGann, Bigge, Spiers, & Seth, 2012; Grant et al., 2016; Nagel, Carl, Kringe, Märtin, & König, 2005; Nau et al., 2013; Siegle & Warren, 2010; Stronks et al., 2016)
Avoidance	Visual-to-auditory	(Borenstein, 1990; Borenstein et al., 2000; Shoval, Borenstein, & Koren, 1998)
	Visual-to-tactile	(Cardin, Thalmann, & Vexo, 2007; Chebat et al., 2018; Dublon & Paradiso, 2012; Grant et al., 2016; Ito et al., 2005; Stronks et al., 2016)
Navigation	Visual-to-auditory	(Borenstein, 1990; Borenstein et al., 2000; Botzer, Shvalb, & Ben-Moshe, 2018; Dunai, Peris-Fajarnés, Lluna, & Defez, 2013; Levy-Tzedek et al., 2012; Shoval et al., 1998; Stoll et al., 2015)
	Visual-to-tactile	(Chebat et al., 2018; Chebat, Schneider, Kupers, & Ptito, 2011; Faugloire & Lejeune, 2014; Grant et al., 2016; Ito et al., 2005; Kupers, Chebat, Madsen, Paulson, & Ptito, 2010; Segond, Weiss, & Sampaio, 2005; Stronks et al., 2016; van Erp, Van Veen, Jansen, & Dobbins, 2005; Zelek, Bromley, Asmar, & Thompson, 2003)
Emotion conveyance	Visual-to-auditory	(Striem-Amit et al., 2012)

Devices which use visual-to-tactile sensory substitution techniques utilise cross-modal pairings, which are more intuitive and analogical than visual-to-auditory sensory substitution techniques. For example, a circle can be directly conveyed on the skin (e.g., on the back or tongue) via tactile cues presented in a two-dimensional circular pattern. To enhance navigation, tactile sensory substitution techniques have been used to represent magnetic North or to provide positional information using a tactile belt or vest (Jones, Nakamura, & Lockyer, 2004; Rochlis, 1998; Visell, 2009). For other examples of visual-to-tactile SSDs, see Tongue Display Unit (Sampaio, Maris, & Bach-y-Rita, 2001), TVSS (Bach-y-Rita, Collins, Saunders, White, & Scadden, 1969; Bach-y-Rita & W. Kercel, 2003), Optacon (Linvill & Bliss, 1966) and Optohapt (Geldard, 1966). Another line of research has manipulated the strength of tactile vibrations to convey distance information that would otherwise be perceived visually. For example, see EyeCane (Maidenbaum, Levy-Tzedek, Chebat, Namer-Furstenberg, & Amedi, 2014), ETA (electronic travel aid) and EOA (electronic orientation aid) (Dakopoulos & Bourbakis, 2010; Farcy et al., 2006; Liu, Liu, Xu, & Jin, 2010), UltraCane and UltraBike (Sound Foresight Technology, 2019a, 2019b). Visual-to-tactile sensory substitution techniques have enabled users to successfully complete a variety of object recognition, localisation, avoidance and navigation tasks (Table 2).

Many approaches have successfully conveyed colour information using cross-modal auditory and tactile feedback. For a detailed review of SoundView, Eyeborg, Kromophone, See ColOr, ColEnViSon, EyeMusic and Creole, see Hamilton-Fletcher and Ward (2013) and Hamilton-Fletcher, Wright, and Ward (2016). For example, EyeMusic utilises the cross-modal correspondences between musical instruments and colour to convey colour information.

**Fig. 3:**
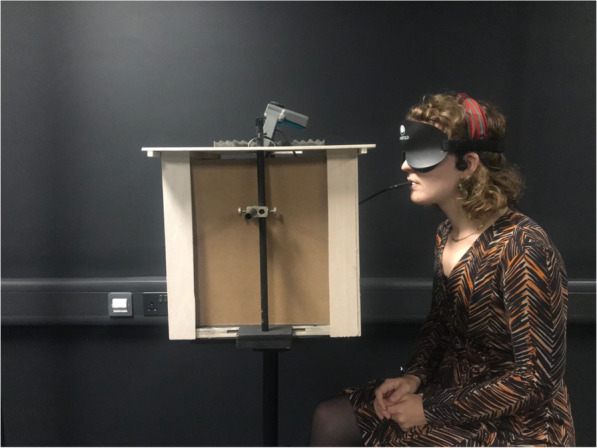
Image showing a user wearing an auditory-tactile cross-modal display prototype. The tactile information is created by BrainPort, a visual-to-tactile sensory substitution device. The auditory information is created by The vOICe, an auditory-to-tactile sensory substitution device. The camera fixated within the box provides real time feed to BrainPort and The vOICe. As a result, the user can perceive the camera feed via audio, tactile and audio-tactile cross-modal feedback

In recent years, a number of cross-modal prototypes that utilise both auditory and tactile feedback have been prototyped and studied in the context of spatial cognition, with encouraging results: EyeCane (Amedi & Hanassy, 2011) and SoV (Hoffmann, Spagnol, Kristjánsson, & Unnthorsson, 2018). In Fig. 3, a low fidelity audio-tactile cross-modal display prototype developed by the authors is also shown. Here, BrainPort (Wicab, 2019) and The vOICe (Meijer, 1992), two commercially available devices that utilise sensory-substitution techniques, are used. BrainPort is a visual-to-tactile sensory substitution device that delivers visual information captured from a live camera via an electro-tactile interface, which is placed on users’ tongues. The vOICe is a visual-to-auditory sensory substitution device that transforms live camera feed into sonifications. Inside the box is a camera connected to these devices. The live camera feed captures an aerial map of multiple targets and delivers this information to the users in tactile, sonification or tactile-sonification forms. Here, the user can acquire spatial information necessary for navigation via electro-tactile stimulation on her tongue and also via sonifications which are delivered by bone conduction headphones (Jicol et al., 2020). The cross-modal display prototype here applies the principles of spatial and temporal coincidence by aligning the sensory information available to the camera. This is simply achieved by fixating the camera with a scaffold inside a box. The box also ensures the consistency of environmental factors for experimentation purposes. The principle of inverse effectiveness enables users to rely equally on the cross-modal feedback from two novel display modes. This reliance is achieved as users have not previously used BrainPort or The vOICe. From a theoretical perspective, this research investigates the relationship between multisensory integration and multisensory combination and how sensory substitution techniques are represented in multisensory processing. From an applied point of view, this research aimed to develop inclusive cross-modal displays that can efficiently deliver the same sensory information in different sensory forms.

Overall, sensory substitution techniques have been prototyped using both modality-specific and cross-modal display modes and have been successfully demonstrated as assistive technologies in a variety of use cases.

### Challenges to widespread adoption of sensory substitution techniques

Despite their documented success in laboratory settings, sensory substitution techniques have not yet gained widespread adoption within the visually impaired population (Chebat et al., 2018). Different groups of researchers offered various explanations for this (Chebat et al., 2018; Spence, 2014; Lenay & Declerck, 2018; Auvray & Farina, 2017; Deroy & Auvray, 2012). They have mainly been criticised for their lack of generalisability beyond the laboratory (Lenay & Declerck, 2018), and some have argued that it is simply implausible that one sense can substitute another (Auvray & Harris, 2014). These arguments, however, are largely aimed at claims that sensory substitution techniques literally substitute a sensory form (i.e., ‘seeing with the brain’ (Bach-y-Rita et al., 1969), ‘seeing with the skin’ (White, Saunders, Scadden, Bach-Y-Rita, & Collins, 1970) or ‘seeing with sound’ (Meijer, 2019).

Approximately 30% of assistive devices are reportedly abandoned before they are even implemented (Phillips & Zhao, 2010). Possible reasons for the abandonment of assistive technologies include the lack of a user-centric approach, difficulty of procurement, poor performance, inability to meet changes in user needs and unaffordable financial costs (Chebat et al., 2018; Phillips & Zhao, 2010). The early abandonment of assistive prototypes arguably has a detrimental impact on individuals with impairments and on wider society. We next explain how adopting an inclusive design mindset when applying sensory substitution techniques could overcome some of the current barriers to the implementation of cross-modal displays for supporting those with disabilities. We also explain how this could lead to further benefits for wider society.

### Sensory substitution techniques as inclusive cross-modal displays

Despite our rich multisensory capabilities within the physical world (Calvert, Spence, & Stein, 2004), relatively few studies have investigated using cross-modal display modes to enhance the ways in which individuals interact with their environments (Sreetharan & Schutz, 2019). Sensory substitution techniques have the potential to transform, extend and augment our perceptual capacities by enabling novel forms of interaction with the environment (Auvray & Myin, 2009; Lenay, Canu, & Villon, 1997; Lenay, Gapenne, Hanneton, Marque, & Genouëlle, 2003). As cross-modal correspondences exist across cultures and languages, they could support a wide range of people, regardless of their capabilities and needs (Jordan & Vanderheiden, 2013). Sensory substitution techniques and cross-modal displays therefore have huge potential to serve different purposes than those served by the assistive technologies described so far.

Extensive research into sensory substitution techniques suggests that various sensory forms (e.g., auditory or tactile) could be utilised interchangeably to have access to the same sensory information (see Table 2). In this way, digital interactions could be made to switch between different senses when delivering the same information. The technologies could be made to be adaptable allowing them to flexibly deliver sensory information depending on user preferences and needs. This would allow sensory substitution techniques to be implemented in a variety of inclusive use cases from extended reality platforms to information and communication applications (Lenay et al., 1997, 2003). Other than assistive technology, a small number of technologies are currently in development which aim to enhance individual’s intact sensory capabilities when their sensory signal strength is temporarily weakened. Cross-modal displays which employ sensory substitution techniques to enhance sensory capabilities are classified as sensory augmentation devices (National Research Council, 2008). The inexpensive application of sensory substitution techniques is possible in the context of sensory augmentation devices with customisable builds and settings (Dublon & Paradiso, 2012). So far, sensory substitution techniques have been applied to firefighters who use tactile gloves equipped with ultrasound sensors when their vision is restricted. These tactile cues provide information about distance, thereby enhancing mobility (Carton & Dunne, 2013). Sensory substitution techniques have also been applied in technologies used by the military, and the alerting systems used in cars to signal an incoming obstacle can be thought of as taking advantage of sensory substitution techniques (Grah et al., 2015; National Research Council, 2008). The potential exists for the use of sensory substitution techniques to benefit a wide range of users.

As sensory substitution stands between perception and cognition (Arnold, Pesnot-Lerousseau, & Auvray, 2017; Esenkaya & Proulx, 2016), exploring sensory substitution phenomena in a broader multisensory context could contribute new insights into how different sensory information and forms are interconnected with each other via cognitive forms. If we can better understand the ways in which sensory substitution techniques work, we may be able to better support individuals who have disabilities by developing inclusive technologies. This could eventually overcome some of the adoption challenges that have been identified with sensory substitution techniques applied as assistive technologies.

Developing a single product or service that appeals to a great number of people is challenging. Inclusive design does not claim to provide omnipotent and omnipresent solutions to address every barrier to usability and accessibility. Instead, the inclusive designer aims to develop flexible and adjustable technologies that appeal to all of us. We suggest this can be done by considering our shared perceptual and/or cognitive capabilities. While individuals vary in terms of their sensory experiences, with some individuals experiencing impairments, more individuals will be similar in their cognitive processing of sensory information. Rather than aim to compensate for impaired sensory forms using specialist devices built for sub-populations, technologies which take advantage of the metamodal organisation of the brain could be used by all individuals.

## Future applications of inclusive cross-modal displays

Sensory substitution techniques have the potential to enable users to alternate between different cross-modal display modes which would allow a wide range of users to access the same device. A simple example could be the way that pedestrians use navigation applications which require frequent screen-dependent feedback. This means of human-computer interaction is more difficult for a pedestrian with a visual impairment, resulting in the development of multiple specialist solutions. While specialist solutions are helpful, an inclusive alternative could co-exist, which would benefit all parties. A navigation application which utilises cross-modal display modes would enable users to switch between auditory and tactile sensory channels as required. The same information will be provided in each of the sensory channels. A visually impaired pedestrian would benefit from this technology since they could receive information about the environment via auditory and/or tactile display modes. Meanwhile, the use of auditory and/or tactile display modes would allow a sighted pedestrian to navigate their environment without relying on visual feedback from a screen, allowing for greater enjoyment of their surroundings. In this way, both users benefit from the use of the same cross-modal displays.

Other future cross-modal displays using sensory substitution techniques could include artistic applications; games; extended reality environments; portable and intuitive systems; and mobility, communication or education platforms (Lenay et al., 1997, 2003; National Research Council, 2008). Sensory substitution techniques can be used to enrich our experiences with the digital world by complementing, and hence reducing, some of the visual information using non-visual cross-modal cues (Hoggan & Brewster, 2007). For example, cross-modal displays could be deployed in conveying emotions via novel sensory forms which do not have a screen dependency, which could improve our tangible interactions with one another.

These examples are all hypothetical. To our knowledge, no such mainstream technologies currently exist which make use of sensory substitution techniques. However, enormous potential exists to develop such cross-modal devices in the future. The scientific literature offers a vast amount of sensory substitution techniques with distinct methods of transforming sensory signals. Investigating their information capacity and perceived resolutions (e.g., Richardson et al., 2019) expands state-of-the-art knowledge regarding multisensory and cross-modal information processing. If these cognitive mechanisms were utilised by applied researchers, the development of innovative technologies which improve access to external information and enhance sensory capabilities of all individuals, regardless of any sensory impairments, would be possible.

### Wider benefits of inclusive cross-modal displays

In recent years, the concepts of cross-modal cognition have spread into multiple disciplines, and examples of their applications can be found in neural networks, artificial intelligence and cognitive robotics (Corradi, Hall, & Iravani, 2017; Hawkins & Blakeslee, 2005; Li, Zhu, Tedrake, & Torralba, 2019; Di Nuovo & Cangelosi, 2015). Now there are new opportunities to make use of sensory substitution techniques in a similar way. These techniques are argued to be ‘universal brain-computer interfaces’ (Danilov & Tyler, 2005) because they make use of the brain’s capacity to inclusively process information, regardless of its original form. In this way, sensory substitution techniques allow us to make sense of information otherwise inaccessible to our natural sensory organs. In this context, sensory substitution techniques can be considered the cognitive transmutation of information to interface. Thinking about sensory substitution in this way brings opportunities to the ways in which we solve modern problems. For example, instead of converting an already existing graphical game (e.g., Pacman or Space Invaders) into an auditory form to improve accessibility for those who are blind, sensory substitution techniques could be utilised to create new forms of multisensory entertainment, to be enjoyed by users with and without visual impairments simultaneously. Why not develop new tools and approaches for novel forms of art (Kim, 2015; Todd Selby, 2011) that can be enjoyed by a wider range of people? Why not focus on multisensory tangible interactions to democratise the ‘pixel empire’ (Ishii, 2019) equally with other senses? Inclusive cross-modal displays have the potential to change how we interact with technology and how technology interacts with us.

## Conclusions

Inclusion is as much about technology, art, policies, social institutions, and commercial models as it is about how one accepts and tolerates others in society. It is a mindset that can be applied in thinking, designing and creating, thereby encouraging all individuals to exist in equilibrium with one another. Overall, these premises offer an inclusive alternative to the usability and accessibility perspectives that are built on a legacy of traditional frameworks, commercial models, and social and academic conversations which view disability as something which accompanies individuals, rather than something which is created by environmental barriers. Human-technology interactions can take advantage of the information processing capability of the metamodal brain in a multisensory context. Rather than creating tools which are merely assistive to compensate for sensory impairments, research and development into sensory substitution techniques could be unified by a motivation for inclusion. New technologies which benefit all individuals could be developed. Accumulated knowledge might then be transferred laterally in a multidisciplinary context, and practically applied to inclusive innovations that appeal to us all.

## Data Availability

Not applicable.

## Abbreviations

ETA: Electronic travel aid
EOA: Electronic orientation aid
HCI: Human computer interactions
TVSS: Tactile vision sensory substitution
SSDs: Sensory substitution devices
